# Assessment of CMR Feature-Tracking Age- and Sex-Dependent Right Ventricular Strain in a Healthy Caucasian Cohort

**DOI:** 10.1007/s12265-024-10557-z

**Published:** 2024-09-18

**Authors:** Jan Eckstein, Hermann Körperich, Oliver M. Weber, Wolfgang Burchert, Volodymyr Pugachov, Oleksandra Demydiuk, Misagh Piran

**Affiliations:** 1 Institut für Radiologie, Nuklearmedizin und Molekulare Bildgebung, Herz- und Diabeteszentrum NRW, Universitätsklinikum der Ruhr-Universität Bochum und Universität Bielefeld Medizinische Fakultät OWL, Bad Oeynhausen, Germany; 2 Philips Clinical Science, Hamburg, Germany

**Keywords:** Cardiac magnetic resonance imaging, Right ventricular strain and strain rate, Normal values, Feature tracking, Gender-dependency, Age-dependency, Reproducibility

## Abstract

Right ventricular (RV) strain offers crucial diagnostic insights in cardiovascular and pulmonary disorders. Nonetheless, the absence of established reference values impedes its clinical implementation. Utilizing CMR-feature tracking, age- and gender-dependent RV strains were systematically assessed in 175 heart-healthy Caucasians, 97 females, median 32.5 years. RV global longitudinal strain (GLS) was greater in females than males (median -26.8% (-28.3;-24.1) vs. -24.4 ± 3.0%; p < 0.001), whereby radial and circumferential strain remained comparable. Age subgroups exhibited increased RV-GLS for group B (30–50 years) (-26.0 ± 3.1% vs. -24.4 ± 3.2%; p = 0.011) and group C (> 50 years) (-26.7 ± 2.3% vs. -24.4 ± 3.2%; p < 0.001) compared to group A (< 30 years). High intra-class correlation coefficients (ICC) were exhibited by intrarater variability (ICC = 0.86–0.95) and moderate levels for interrater variability (ICC = 0.50–0.73). CMR-feature tracking provides a fair quantification method of age- and gender-specific normal RV strain values, demonstrating that higher RV-GLS is linked to female gender and advancing age within a healthy Caucasian cohort.

**Graphical Abstract:**

Right-ventricular global longitudinal strain, assessed by cardiac MRI feature-tracking, increases with the female sex and advancing age within a Caucasian cohort of healthy subjects (N = 175)

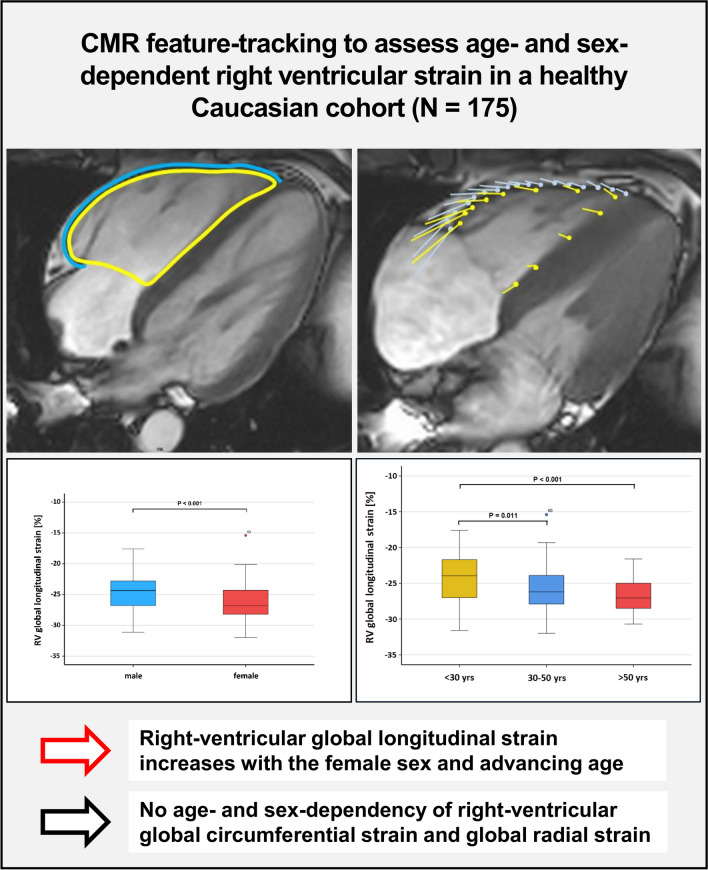

## Introduction

There has been growing clinical application of right ventricular (RV) strain in a broad spectrum of cardiovascular and pulmonary conditions, per example for idiopathic pulmonary arterial hypertension, right heart failure, arrhythmias and hypertrophic phenotypes [1–4]. However, their use is limited by the absence of established normal right ventricular strain values essential for reliable detection of clinical or even subclinical alterations. Left ventricle strains have been consolidated in larger cohort studies [5–8], whereby right ventricular strain has received lesser acknowledgement. Additionally, the studies that recently published normal reference values for CMR quantified right ventricular strains present various data incongruencies [9–12], further underscoring the need for systematic analysis.

Echocardiographic assessment of right ventricular function remains technically challenging due to the dense myocardial trabeculae, its location behind the sternum with intricate anatomical features and the complexity of its motion patterns [13, 14]. Moreover, modality-dependent limitations of echocardiography comprise the acoustic window limitations, operator-dependence as well as patient echogenicity. Given these constraints, cardiac magnetic resonance imaging (CMR) has become the established gold standard for evaluation of the right ventricle [15–17], providing high resolution multiparametric and reproducible cardiac function analysis. Feature tracking (FT) represents an established CMR technique for strain quantification via tracking of myocardial points throughout the cardiac cycle using a specialized algorithm [9, 18].

While a recent large Asian-cohort study conducted by Li et al. [9] contributes to the existing body of research, our study stands out for the systematic CMR right ventricular strain assessment in a large Caucasian cohort. This cohort has been segregated by age and gender and assessed for reproducibility, offering a comprehensive analysis that enriches our understanding of physiological adaptations within the Caucasian population. Consequently, we hypothesize the presence of significant differences in strain across different age and gender groups and high levels of reproducibility observed in CMR measurements.

Despite the paramount importance of customizing diagnostic approaches to suit individual patient characteristics, the integration of age- and gender-specific variations into right ventricular assessments has largely been overlooked in clinical practice. This oversight underscores a substantial gap in patient-specific diagnostics, which could significantly impact the accuracy and effectiveness of medical care.

## Methods

### Study Population

This is a retrospective, single-center, cross-sectional study involving the recruitment of 208 volunteers via a public call spanning from September 2017 to December 2020. Ethical approval was obtained from the local ethics committee (Ethikkommission der Medizinischen Fakultät der Ruhr-Universität Bochum, Sitz Ostwestfalen, registration number: 2017–238, with an amendment registration number 2022–924), adhering to the principles outlined in the Declaration of Helsinki's seventh revision of 2013. Prior to participation, all volunteers or their legal guardians provided informed consent.

Participants underwent a comprehensive health questionnaire to select only healthy individuals, devoid of cardiovascular diseases, personal or familial cardiac history, and associated risk factors, were included in the study. Demographic information including age, gender, weight, and height was collected from the questionnaire. Additionally, it was ensured that no contra-indications for CMR assessment were present. Routine biventricular CMR assessments, encompassing left and right ventricular function parameters such as ejection fractions and left ventricular muscle mass, were conducted to validate data consistency against established norms [5].

**Fig. 1:**
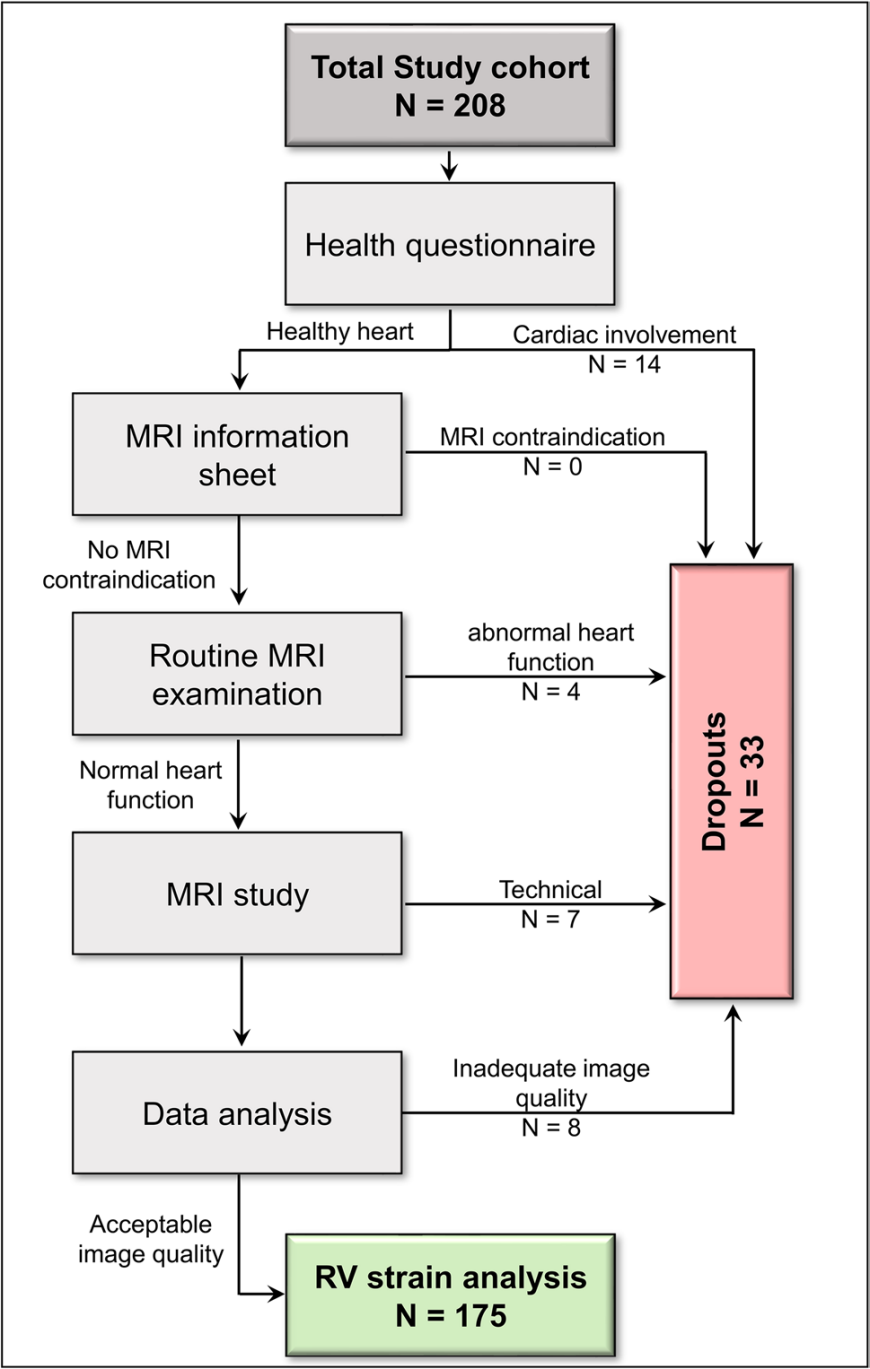
Flow chart to identify healthy study participants suitable for performing right-ventricular strain analysis

Eighteen participants were excluded due to health criteria violations, while technical constraints or inadequate image quality led to the exclusion of fifteen additional participants (Fig. 1).

### Cardiac MRI

All participants underwent cardiac MRI imaging utilizing a 3.0 Tesla multi-transmit magnetic resonance imaging system (Achieva, Philips Healthcare, Best, The Netherlands; Release 5.3.1 and 5.6.1), which incorporates advanced dStream technology. Cardiac cine acquisitions were performed using vector electrocardiogram triggering, ensuring precise synchronization. The system boasted a maximum gradient performance of 40 mT/m with a slew rate of 200 mT/m/ms, while signal reception was facilitated by cardiac phased-array coils.

Our imaging protocol comprised an axially acquired stack covering the entire heart, alongside a short-axis stack encompassing both left and right ventricles (typically 12–16 slices, with no gaps) as well as 2-chamber, 3-chamber and 4-chamber views according to the guidelines, employing cine steady-state free-precession acquisitions (TR/TE/flip angle = 2.7 ms/1.35 ms/42°). Volumetric and RV strain assessment were enabled utilizing both 4-chamber long-axis and short-axis views. With a rapid acquisition rate, 45 reconstructed heart frames (interpolated, 32 acquired cardiac phases) were captured within a single cardiac cycle, ensuring a temporal resolution of < 30 ms. Spatial resolution was optimized at 1.5 × 1.5 × 8 mm^3^, facilitating precise anatomical delineation and accurate assessment of cardiac function. All examinations were conducted by a single investigator in order to minimize subjective interactions.

### Strain Analysis

Strain analysis was conducted using the CVI42® software package (Circle Cardiovascular Imaging Inc., Calgary, Canada, Release 5.12.1) based on cine steady-state free-precession acquisitions. The 4-chamber and short axis views were utilized for right ventricular strain assessment. The RV strain assessment was carried out by two experienced evaluators with > 3 years of experience.

Briefly, after loading the patient data, the Dubois formula was selected for the calculation of the body-surface-indexed ventricular volumes. Before starting the strain analysis, the “Use simplified endocardial contours” option under the *preferences* tab, subfolder *Contours*, and the “Apply a temporal smoothing” option under the subfolder *Strain*, were activated. Out of the typically acquired 3–5 four-chamber slices, the most suitable 4-chamber slice, showing the largest RV-extension, was selected for RV strain analysis.

**Fig. 2:**
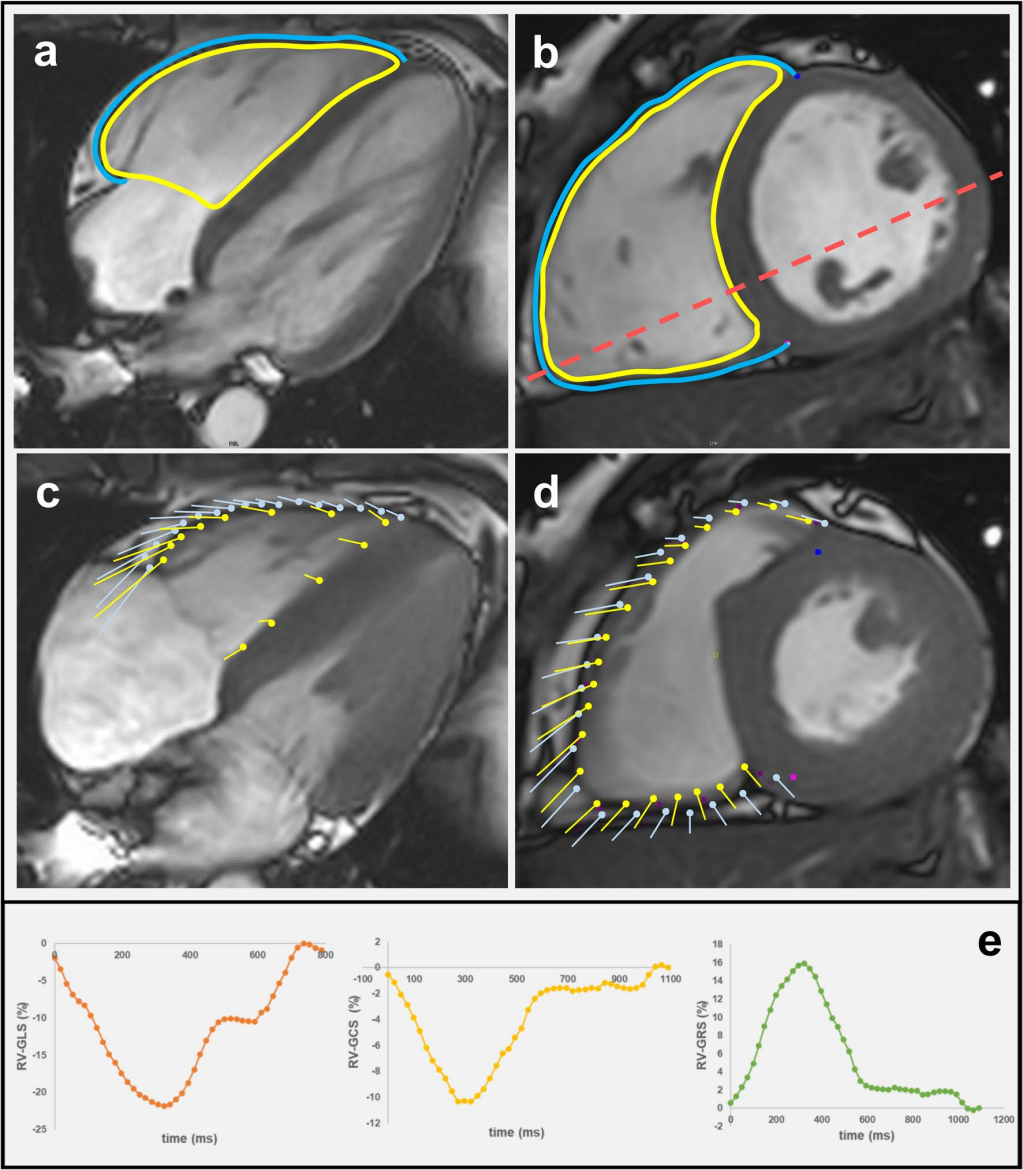
Illustration of slice-selection in the 4-chamber view at end-diastole (red dashed line) in order to capture the maximum dimension of right ventricular (RV) wall deformation. (**a**) 4-Chamber view (**b**) short axis view. Yellow lines represent the borders of the right endocardium, blue lines correspond to the borders of the right epicardium. (**c**, **d**) Boundary points to visualize displacement, direction and vector length for calculating of the cardiac strain. (**e**) Time-to-strain curves displaying the RV global longitudinal strain (orange), RV global circumferential strain (yellow), and RV global radial strain (green)

To perform the RV strain analysis, both the short axis (SA) stack and the previously selected, most suitable 4-chamber slice were loaded into the CVI42® strain module (Fig. 2). Subsequently, the appropriate end-systolic and end-diastolic heart frames were determined. Using the option “Detect SAX contours current phase” for the SA stack and the option “Contours current 4 CV image” for the relevant 4-chamber slice, the CVI42® software automatically drew contours for these two heart frames. To improve the reliability of the RV strain analysis, the basal SA slices representing the right ventricular outflow track and the apical SA slices with no obvious RV blood volume in end-systole were excluded from RV strain analysis. If needed, manual correction of the endocardial SA contours and the endocardial 4-chamber contours was made. The epicardial contours for both the SA stack and the selected 4-chamber slice had to be precisely drawn manually. RV strain analysis was initiated based on an end-diastolic heart frame. If required, the end-systolic and end-diastolic heart frames must be readjusted. This is because the software can only calculate the end-systolic and end-diastolic volumes based on the remaining slices due to the deletion of some SA slices in the previously described step, resulting in a wrong definition of the heart frames for end-systole and end-diastole.

The RV strain results for global longitudinal strain, global circumferential strain, and global radial strain should then be carefully checked. This can be done by displaying the strain results in cine mode by selecting the CVI42® options “Boundary points” and/or “Mesh”. Finally, the strain data (“scientific report”) is saved for reporting and export as a text file for later extraction of the relevant data (strain and strain rate values, time-to-peak data, displacement data etc.) into the database.

Alongside volumetric data, peak systolic and diastolic longitudinal, circumferential and radial strain and strain rate was quantified.

### Statistics

Statistical analysis was conducted using SPSS (version 29.0, IBM Deutschland GmbH). Normal distribution of data was assessed using the Shapiro–Wilk test. Continuous variables were reported as mean ± standard deviation (SD) for normally distributed data, while non-normally distributed data were presented as median with interquartile range. Gender differences were evaluated using the Mann–Whitney U-test for non-normally distributed data and the unpaired Student’s t-test for normally distributed data. Correlations between right ventricular strain and strain rate with age was assessed using either the Spearman or Pearson correlation coefficient, depending on the fulfillment of necessary assumptions. Prior to conducting Pearson or Spearman’s Rho correlation analysis, linearity, normal distribution, and the presence of outliers were carefully evaluated. The relationship between > 2 predictors and a dependent variable was determined by multilinear regression analysis. The analysis was only accepted if the prerequisites for conducting a multilinear regression analysis, including linearity, checking for outliers, independence of residuals, multicollinearity, homoscedasticity and normal distribution according to Hemmerich**,** were met [19]. The p-value < 0.05 was considered statistically significant. The correlation coefficient (r resp. rho) was interpreted according to Cohen's guidelines [20]. A strong correlation was defined for r resp. rho values above 0.5, a moderate correlation for values between 0.3 and 0.5, and a weak correlation for values between 0.1 and 0.3. Moreover, right ventricular strains were assessed against age-segments, divided in groups of subjects below 30 years, subjects between 30 – 50 years and subjects above 50 years of age. The intra- and interrater reliability was examined utilizing both the intraclass correlation coefficient (ICC), coefficient of variation and Bland–Altman statistics.

## Results

### Baseline Characteristics

This study enrolled 175 healthy Caucasian subjects, comprised of 78 males and 97 females. The median age with interquartile range marked 32.5 {21.4; 48.6} years and was found comparable between both genders (p = 0.763). Males presented increased body mass index (BMI) (24.6 ± 3.8 vs. 22.5 {20.5; 24.5}; p < 0.001) compared to females. Details on baseline demographic data is summarized in Table 1.

**Table 1:** Baseline characteristics

	all	male	female	P-value
n	175	78	97	
age (years)	32.5 {21.4; 48.6}	32.0 {20.7; 49.8}	34.3 {22.2; 48.3}	0.763
weight (kg)	68 {60; 81}	82 ± 15	61 {57; 68}	** < 0.001**
height (cm)	173 ± 12	182 ± 8	166 {160; 171}	< 0.001*
body surface area (m^2^)	1.84 ± 0.25	2.03 ± 0.21	1.69 ± 0.16	** < 0.001**
body mass index (kg/m^2^)	23.2 {20.9; 26.1}	24.6 ± 3.8	22.5 {20.5; 24.5}	0.001*
Physical activity		athletic sports	endurance sports	miscellaneous
	N	(%)	(%)	(%)
Several times per week	78	26.8	70.7	2.4
Once per week	48	6.9	72.4	20.7
2–3 per month	19	14.3	57.1	28.6
never	30	-	-	-

Data reported as mean ± standard deviation or median (interquartile range). * Mann–Whitney-U-test otherwise unpaired Student’s t-test; n, number of subjects

### Gender-Divided Biventricular Volumetrics and Pulmonary Artery Hemodynamics

Males presented significantly enlarged biventricular volumetrics in contrast to females, per example reflected by their indexed end diastolic volume (RV: 86.2 ± 9.7 ml/m^2^ vs. 74.4 ± 11.3 ml/m^2^; p < 0.001, LV: 80.0 ± 8.7 ml/m^2^ vs. 72.3 ± 8.8 ml/m^2^; p < 0.001). Notably, females compared to males showed decreased indexed pulmonary artery stroke volume (49.7 ± 6.9 ml vs. 52.0 ml ± 6.6; p = 0.017) and lower PA peak velocities (p < 0.001). On the other hand, females compared to males presented higher right ventricular ejection fractions (63% {57; 65}; 59 ± 5%; p < 0.001), increased left ventricular ejection fraction (64 ± 5% vs. 67 ± 5%; p = 0.002) alongside increased left ventricular global longitudinal, circumferential and radial strains (p < 0.001). Further parameters, characterizing diastolic function and pulmonary artery elasticity remained statistically comparable between both genders. Additional details on gender-divided biventricular volumetrics and pulmonary artery hemodynamics are presented in Table 2.

**Table 2:** Gender-divided biventricular volumetrics and pulmonary artery hemodynamics

	all	male	female	P-value
Heart rate (bpm)	65 {59; 73}	64 {57; 72}	66 {60; 73}	0.147
RV EDV_i_ (ml/m^2^)	79.7 ± 12.1	86.2 ± 9.7	74.4 ± 11.3	** < 0.001**
RV ESV_i_ (ml/m^2^)	31.0 {26.2; 36.8}	35.5 ± 6.5	27.8 {23.8; 31.9}	< 0.001*
RV SV_i_ (ml/m^2^)	47.9 ± 7.1	50.7 ± 6.7	45.6 ± 6.7	** < 0.001**
RV ejection fraction (%)	60 ± 5	59 ± 5	63 {57; 65}	< 0.001*
LV EDV_i_ (ml/m^2^)	75.5 ± 9.6	80.0 ± 8.7	72.3 ± 8.8	< 0.001*
LV ESV_i_ (ml/m^2^)	26.1 ± 5.4	28.6 ± 5.1	24.2 ± 4.9	** < 0.001**
LV SV_i_ (ml/m^2^)	49.4 ± 6.7	51.4 ± 6.6	48.0 ± 6.3	** < 0.001**
LV ejection fraction (%)	66 ± 5	64 ± 5	67 ± 5	**0.002**
LV GLS (%)	-16.9 ± 1.8	-15.9 ± 1.5	-17.6 ± 1.6	< 0.001*
LV GCS (%)	-19.3 ± 2.1	-18.1 ± 1.7	-20.1 ± 1.9	** < 0.001**
LV GRS [%]	33.5 {29.7; 37.7}	30.8 ± 4.6	36.5 {32.7; 39.6}	** < 0.001**
PA SV_i_ (ml)	50.7 ± 6.8	52.0 ± 6.6	49.7 ± 6.9	0.017*
PA V_max_ (cm/c)	85 {80; 100}	95 {85; 105}	80 {75; 90}	< 0.001*
PA CI (L/min/m^2^)	3.35 {2.90; 3.70}	3.40 {2.90; 3.80}	3.32 ± 0.52	0.705*
MAPSE (cm)	1.7 ± 0.3	1.7 ± 0.3	1.6 ± 0.3	0.170
TAPSE (cm)	2.3 {2.0; 2.5}	2.3 ± 0.4	2.3 {2.0; 2.5}	0.594*
PV S/D (a.u.)	1.1 {0.9; 1.4}	1.1 ± 0.4	1.1 {0.9; 1.4}	0.712*
MV E/A (a.u.)	1.9 {1.6; 2.4}	1.9 ± 0.6	1.9 {1.6; 2.4}	0.728*
Relative area change (%)	36.4 ± 10.0	37.5 ± 9.8	35.1 ± 10.0	0.076
PA/AO ratio (a.u.)	1.1 ± 0.2	1.1 ± 0.2	1.1 ± 0.2	0.553
PA elasticity (%)	57.6 {41.1; 78.7}	59.2 {44.6; 81.4}	55.2 {37.9; 73.8}	0.115*
PA beta stiffness index (a.u.)	1.45 {1.06; 2.03}	1.39 {1.00; 1.84}	1.52 {1.13; 2.21}	0.090*

Data reported as mean ± standard deviation or median (interquartile range). * Mann–Whitney-U-test otherwise unpaired Student’s t-test; AO ascending aorta; CI cardiac index; E/A ratio E-wave to A-wave; EDV_i_, BSA-indexed enddiastolic volume; ESV_i_, BSA-indexed endsystolic volume; SV_i_, BSA-indexed stroke volume; GLS, global longitudinal strain; GCS, global circumferential strain; GRS, global radial strain; LA, left atrial; LV left ventricular; PA pulmonary artery; RV, right ventricular; MAPSE mitral annulus plain systolic excursion; MV mitral valve; PV right upper pulmonary vein; S/D ratio S-wave to D-wave; TAPSE, tricuspidal annulus plain systolic excursion; V_max_ peak velocity

### Gender-Divided Right Ventricular Strain and Strain Rates

**Fig. 3:**
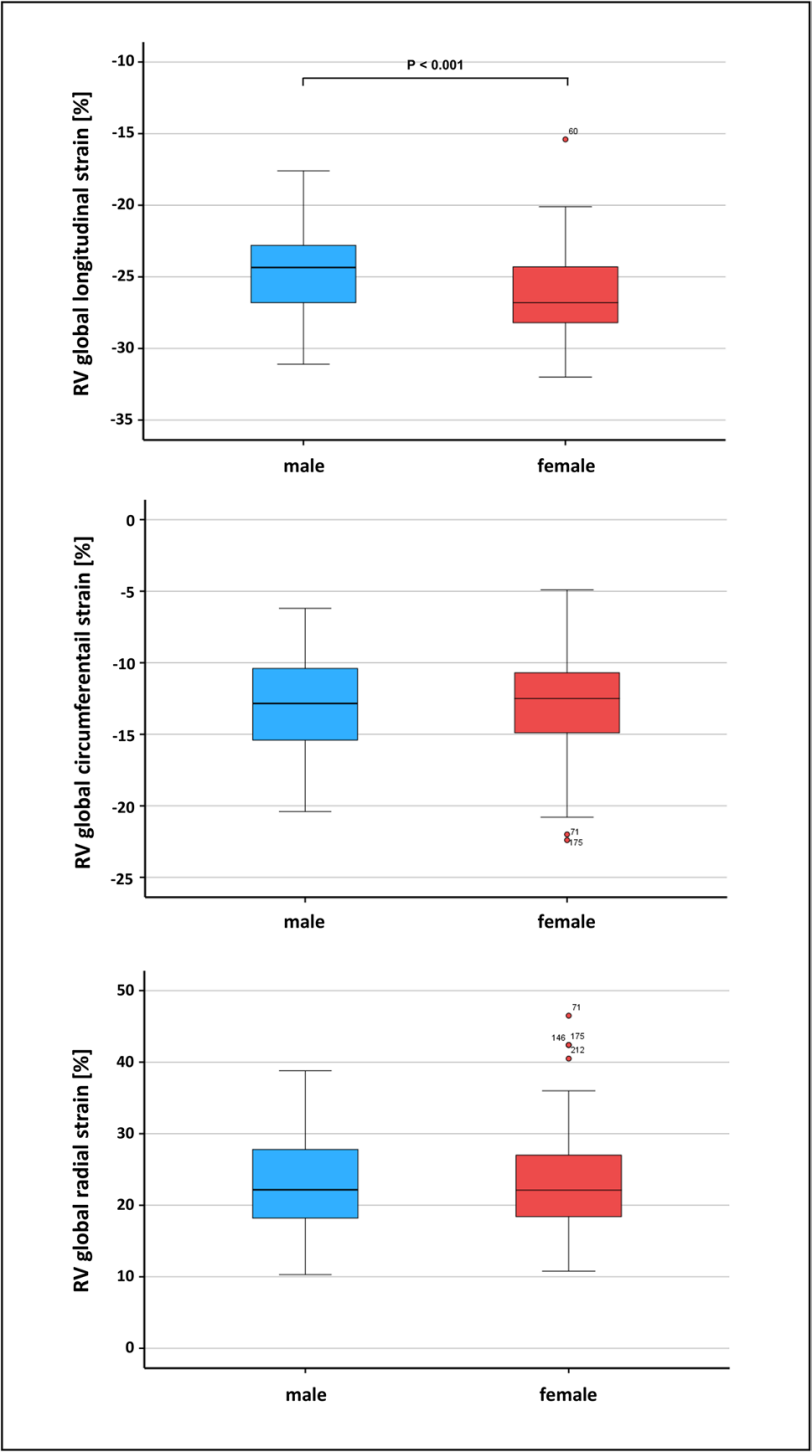
Box plots showing the sex-specific differences in right ventricular global strains

Global longitudinal right ventricular strain was found significantly greater in females than males (-26.8 {-28.3; -24.1} % vs. -24.4 ± 3.0%; p < 0.001), whereas radial (p = 0.963) and circumferential (p = 0.980) strain remained comparable (Fig. 3). However, females compared to males presented increased time to peak (ms) in radial, circumferential and longitudinal dimensions (p = 0.002–0.035). Except for diastolic global longitudinal strain rate which is greater for females than males (1.48 {1.26; 1.76} vs. 1.30 ± 0.24; p < 0.001), all other remaining strain rates were found comparable between both genders. Significant alterations in displacement were found in the radial dimension, displaying increased displacement for males compared to females (4.5 ± 0.76 mm vs. 4.2 {3.6; 4.8} mm; p = 0.026). Further gender-divided right ventricular strain data is summarized in Table 3.

**Table 3:** Gender-divided right ventricular strain and strain rate

	all	male	female	P-value
RV-GRS (%)	22.1 {18.2; 27.2}	22.6 ± 5.6	22.1 {18.3; 27.0}	0.963*
RV-GCS (%)	-12.9 ± 3.1	-12.9 ± 2.9	-12.5 {-15.0; -10.7}	0.980*
RV-GLS (%)	-25.5 ± 3.1	-24.4 ± 3.0	-26.8 {-28.3; -24.1}	< 0.001*
TTP radial (ms)	317 {295; 332}	308 {290; 328}	323 ± 27	0.002*
TTP circumferential (ms)	316 {296; 337}	307 {291; 329}	323 ± 29	0.007*
TTP longitudinal (ms)	330 {311; 353}	326 {304; 347}	336 ± 30	0.035*
RV-GRSR systolic (1/s)	1.03 {0.85; 1.28}	1.07 {0.86; 1.35}	0.99 {0.82; 1.24}	0.249*
RV-GCSR systolic (1/s)	-0.63 {-0.79; -0.53}	-0.63 {-0.80; -0.54}	-0.62 {-0.76; -0.51}	0.389*
RV-GLSR systolic (1/s)	-1.23 {-1.46; -1.08}	-1.21 {-1.45; -1.07}	-1.28 {-1.50; -1.08}	0.332*
RV-GRSR diastolic (1/s)	-1.19 {-1.50; -1.03}	-1.16 {-1.47; -0.99}	-1.24 {-1.58; -1.06}	0.104*
RV-GCSR diastolic (1/s)	0.68 {0.56; 0.80}	0.66 {0.53; 0.77}	0.70 {0.59; 0.82}	0.080*
RV-GLSR diastolic (1/s)	1.39 {1.20; 1.64}	1.30 ± 0.24	1.48 {1.26; 1.76}	< 0.001*
Radial displacement (mm)	4.3 {3.8; 4.9}	4.5 ± 0.76	4.2 {3.6; 4.8}	0.026*
circumferential displacement (mm)	0.8 {-2.1; 1.8}	0.8 {-1.7; 1.6}	1.1 {-2.2; 1.9}	0.611
longitudinal displacement (mm)	11.6 ± 3.3	11.4 ± 3.9	11.7 ± 2.6	0.542

Data reported as mean ± standard deviation or median (interquartile range). * Mann–Whitney-U-test otherwise unpaired Student’s t-test; GLS, global longitudinal strain; GCS, global circumferential strain; GRS, global radial strain; GLSR, global longitudinal strain rate; GCSR, global circumferential strain rate; GRSR, global radial strain rate; RV, right ventricular; TTP time-to-peak

### Correlations Between Right Ventricular Strains and Age

Advancing age is associated with a moderate increase in right ventricular global longitudinal strain (r = -0.316), further reflected by its corresponding measure of displacement (r = 0.415). Moreover, advancing age results in significant alterations for radial, circumferential and longitudinal dimensions of displacement (p = < 0.001 – 0.041). With the exception of diastolic longitudinal strain rate, undergoing significant alterations with advancing age (p = 0.036), the remaining strain rates present no statistically significant variations in association with age. Further correlations are summarized in Table 4.

**Table 4:** Correlations between right ventricular strains and age

	r resp. rho	P-value
RV-GRS (%)	-0.067	0.380*
RV-GCS (%)	0.126	0.098*
RV-GLS (%)	-0.316	< 0.001*
TTP radial (ms)	0.287	< 0.001*
TTP circumferential (ms)	0.303	< 0.001*
TTP longitudinal (ms)	0.137	0.072*
RV-GRSR systolic (1/s)	-0.135	0.075*
RV-GCSR systolic (1/s)	0.098	0.198*
RV-GLSR systolic (1/s)	-0.119	0.118*
RV-GRSR diastolic (1/s)	0.127	0.095*
RV-GCSR diastolic (1/s)	-0.104	0.171*
RV-GLSR diastolic (1/s)	0.159	0.036*
Radial displacement (mm)	0.172	0.022*
circumferential displacement (mm)	-0.154	**0.041**
longitudinal displacement (mm)	0.415	< 0.001*

^*^ Spearman’s Rho correlation otherwise Pearson product-moment correlation. Interpretation according to Cohen (1988), weak correlation = 0.10, moderate correlation = 0.30, strong correlation = 0.50 (bold)

GLS, global longitudinal strain; GCS, global circumferential strain; GRS, global radial strain; GLSR, global longitudinal strain rate; GCSR, global circumferential strain rate; GRSR, global radial strain rate; RV, right ventricular; TTP time-to-peak

### Age-Subgroup-Divided Right Ventricular Strains

**Fig. 4:**
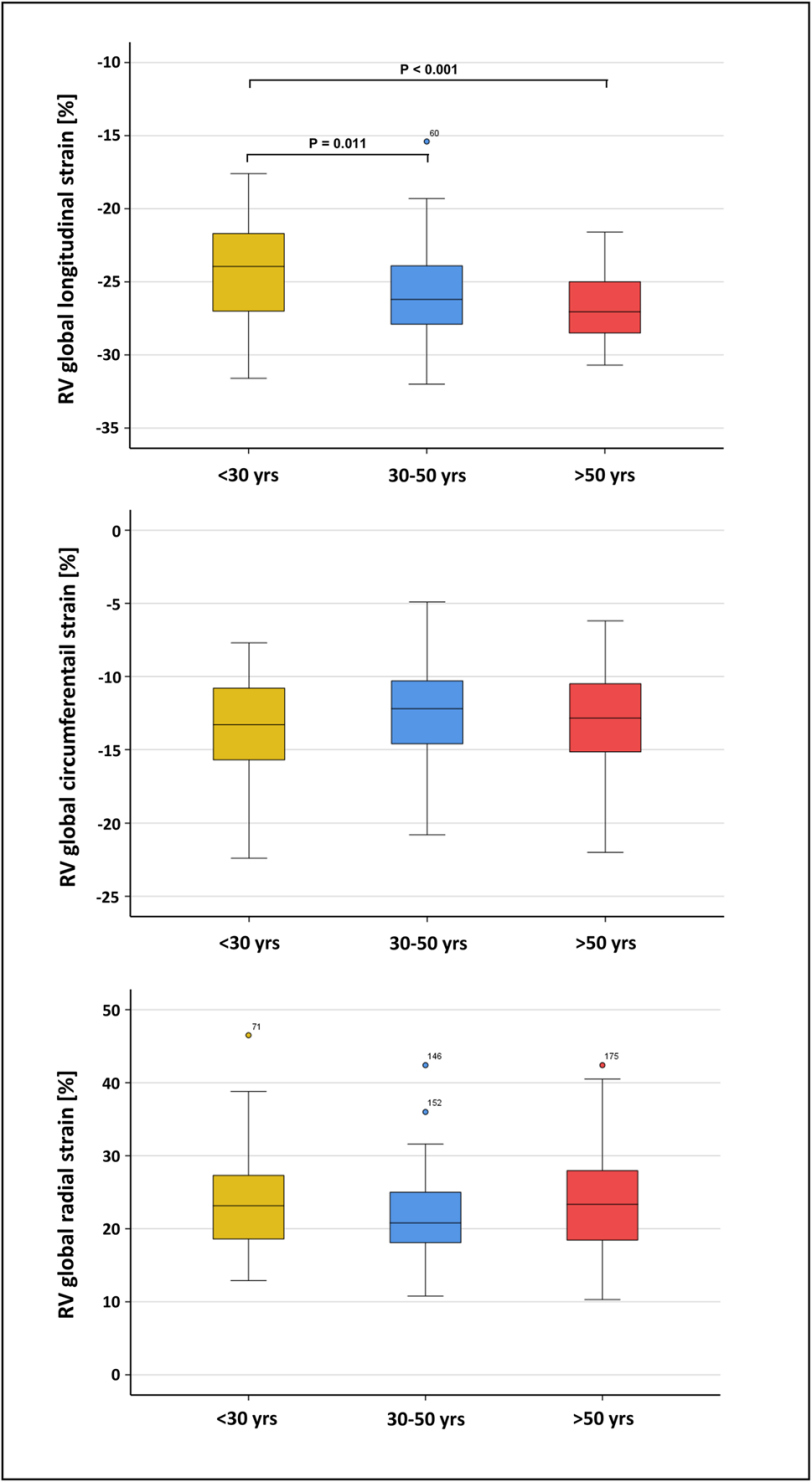
Box plots showing the age-specific differences in right ventricular global strains found in the three subgroups

Age-subgroups presented statistically significantly increased global longitudinal right ventricular strains between group A (< 30 years) and group B (30 – 50 years) (-24.4 ± 3.2% vs. -26.0 ± 3.1%; p = 0.011) as well as between group A and group C (> 50 years) (-24.4 ± 3.2% vs. -26.7 ± 2.3%; p < 0.001, see Fig. 4). A similar change was found for longitudinal displacement, with significantly greater displacement among subjects of group C or group B compared to group A (p < 0.001). Radial and circumferential global right ventricular strains presented no statistical significance. However, radial and circumferential time to peak was found significantly elevated for the older subjects of group C compared to the youngest subjects of group A (p < 0.001), whereas longitudinal time to peak showed no statistical significant alteration. Overall strain rates showed no statistically significant alterations between the age-subgroups. Further details are summarized in Table 5.

**Table 5:** Age-subgroup-divided right ventricular strains

	A (< 30 yrs)	B (30–50 yrs)	C (> 50 yrs)	Comparison	Post-hoc test*
N	74	61	40		
RV-GRS (%)	23.2 {18.6; 27.4}	20.8 {18.0; 25.8}	23.8 ± 7.3		P = n.s
RV-GCS (%)	-13.3 ± 2.9	-12.4 ± 3.0	-13.1 ± 3.4		P = n.s
RV-GLS (%)	-24.4 ± 3.2	-26.0 ± 3.1	-26.7 ± 2.3	A – B A – C B – C	**P = 0.011** **P < 0.001** P = 0.566
TTP radial (ms)	309 {288; 327}	317 ± 24	331 ± 32	A – B A – C B – C	P = 0.240 **P < 0.001** P = 0.064
TTP circumferential (ms)	308 {287; 324}	319 ± 25	331 ± 28	A – B A – C B – C	P = 0.107 **P < 0.001** P = 0.115
TTP longitudinal (ms)	324 {305; 345}	334 ± 33	335 ± 28		P = n.s
RV-GRSR systolic (1/s)	1.12 {0.89; 1.37}	0.91 {0.82; 1.21}	1.08 ± 0.34		P = n.s
RV-GCSR systolic (1/s)	-0.65 {-0.85; -0.55}	-0.61 {-0.74; -0.50}	-0.66 ± 0.17		P = n.s
RV-GLSR systolic (1/s)	-1.21 {-1.42; -1.04}	-1.23 {-1.51; -1.08}	-1.28 {-1.56; -1.10}		P = n.s
RV-GRSR diastolic (1/s)	-1.24 {-1.60; -1.06}	-1.17 {-1.46; -0.99}	-1.24 ± 0.39		P = n.s
RV-GCSR diastolic (1/s)	0.70 {0.58; 0.81}	0.67 {0.54; 0.78}	0.67 {0.53; 0.82}		P = n.s
RV-GLSR diastolic (1/s)	1.33 {1.12; 1.53}	1.44 ± 0.29	1.49 ± 0.37		P = n.s
Radial displacement (mm)	4.2 ± 0.73	4.3 ± 0.79	4.6 {4.0; 5.5}	A – B A – C B – C	P = 0.627 **P = 0.012** P = 0.263
circumferential displacement (mm)	1.2 {-1.9; 2.0}	0.8 {-2.1; 1.6}	-1.3 {-2.2; 1.5}		P = n.s
longitudinal displacement (mm)	10.1 ± 2.9	12.5 ± 3.2	13.0 ± 2.8	A – B A – C B – C	**P < 0.001** **P < 0.001** P = 1.000

Data reported as mean ± standard deviation or median (interquartile range). *Kruskal–Wallis-Test as global test with Mann–Whitney U-test for pairwise comparisons with Bonferroni correction; GLS, global longitudinal strain; GCS, global circumferential strain; GRS, global radial strain; GLSR, global longitudinal strain rate; GCSR, global circumferential strain rate; GRSR, global radial strain rate; RV, right ventricular; TTP time-to-peak

### Multiple Linear Regression Analysis

Based on the previously performed bivariate correlation analyses, the parameters sex, age and diastolic right ventricular longitudinal strain rate (RV-GLSR _diastolic_) were tested to evaluate their contribution to global longitudinal right ventricular strain. The R^2^ for the overall model was 0.435 (adjusted R^2^ = 0.425), indicative for a high goodness-of-fit according to Cohen [19]. All three independent variables were able to predict RV-GLS statistically significantly (age, p < 0.001; sex, p = 0.023; RV-GLSR _diastole_ p < 0.001) with F(3, 169) = 43.328, p < 0.001 according to the following equation:

$$RV-GLS=-0.047*age-0.865*sex-4.924*RV-{GLSR}_{diastole}-16.411$$

For better comparability, all coefficients were additionally standardized to replace units with standard deviations. The calculated beta coefficients for age were -0.235, sex -0.142 and RV-GLSR _diastole_ -0.515.

### Intra- and Interrater Reliability in Global Right Ventricular Strain

Intra- and interrater variability was examined for 20 randomized subjects for global longitudinal, circumferential and radial right ventricular strains. Intrarater variability resulted in good (0.75–0.90) to excellent (> 0.90) intra-class-correlation coefficients (ICC = 0.86 to 0.95) [21] with low variability (CoV < 10%). However only moderate (0.50 – 0.75) intra-class-correlation coefficients for interrater variability was achieved (ICC = 0.50 to 0.73) with comparatively increased variability (CoV = -8.5 to 17.4%). For both intra- and interrater analyses global longitudinal strains showed lowest variability among all three strain types. Further details are presented in Table 6.

**Table 6:** Intra- and interrater reliability in global right ventricular strain (N = 20)

	Mean difference [%] (limits of agreement)	ICC	CoV [%]
intrarater			
RV-GRS	0.3 (-3.5 to 4.2)	0.95	5.2
RV-GCS	-0.1 (-2.1 to 1.9)	0.95	-4,9
RV-GLS	0.7 (-2.4 to 3.8)	0.86	-3,6
interrater			
RV-GRS	2.5 (-8.6 to 13.6)	0.72	17.4
RV-GCS	-1.1 (-6.3 to 4.1)	0.73	-13.2
RV-GLS	-2.2 (-9.1 to 4.7)	0.50	-8.5

CoV, coefficient of variation; ICC, intra-class correlation coefficient; GRS, global radial strain; GCS, global circumferential strain; GLS, global longitudinal strain; RV, right ventricular

## Discussion

The diagnostic and prognostic potential of CMR-derived right ventricular strain has been extensively investigated across various pathological conditions, such as myocarditis [22] and chronic thromboembolic pulmonary hypertension [23]. However, its clinical utility is hampered by the lack of established normal values. To the best of our knowledge, this study is the largest Caucasian cohort study, addressing this gap by presenting CMR derived right ventricular strain values stratified by age and gender. The present study presents the following findings:

- Females present significantly elevated right ventricular global longitudinal strain in contrast to males
- Global longitudinal strain increases with advancing age
- CMR intra- and interrater variability analysis consolidates reliable levels of reproducibility, particularly regarding right ventricular circumferential and radial strain quantification

The significantly elevated right ventricular global longitudinal strain of females in contrast to males observed in the present study is in line most with larger cohort-sized CMR [9, 12] and echocardiographic [24, 25] study findings. This gender-related differences in global longitudinal strain most likely results from the volumetric differences between males and females, as males exhibit higher values of indexed right ventricular end-diastolic volume, indexed right ventricular end-systolic volume and lower right ventricular ejection fraction.

However, the present CMR study data is not consistent with findings of elevated global circumferential and radial strain in females in contrast to males [9, 26]. Contrasting these findings, Peng et al. found no gender differences when quantifying right ventricular longitudinal and radial strains [11]. The data incongruencies displayed by the various published CMR normal reference may be of technical nature. Currently, there is no detailed, established procedure for right ventricular strain assessment. Per example incorporation of the 3-chamber view, contouring of the interventricular septal wall, the number of slices utilized in each axis represent only a handful of possible factors that may ultimately alter strain values significantly. Moreover, all comparable studies were Asian-cohort based, suggestive of possible ethnic differences in wall deformation. Controversial findings further comprised circumferential, radial and longitudinal strain rates, with none or some statistical significant differences found by Qu et al. [12] and Liu et al. [26], whereas solely statistical significant differences were found by Li et al. [9], challenging the diagnostic value of these parameters as well as their replicability. Their poor clinical utility was further consolidated by recent literature investigating diagnostic strain rate value for hypertrophic phenotype discrimination [3, 27].

Overall, global longitudinal right ventricular strain appears of primary clinical importance, when considering gender-specific patient diagnostics.

The study results present a rise in global longitudinal strain with age, aligning with the findings of Li et al. [9]. This is of clinical importance as longitudinal shortening constitutes the primary mechanism of RV contraction, with RV function closely tied to this aspect [28]. Assessments of longitudinal strain hold potential for detection of subclinical RV dysfunction prior to the manifestation of abnormalities such as changes in right ventricular ejection fraction or stroke volume [29]. The elevation in longitudinal strain with age could be a compensatory mechanism in heart-healthy older subjects attributed to subclinical fibrotic remodeling and chamber dilation. The age-associated diastolic function decline is closely coupled to left atrial hemodynamics [30]. The ageing-associated reduction in ventricular compliance will consecutively demand a greater active contractile force to eject blood at end-systole, reflected in increased longitudinal wall deformation. These signs of age-dependent cardiac remodeling may further contribute towards the altered velocity–time profiles in the pulmonary artery hemodynamics recently described [31].

Li et al. [9] and Liu et al. [26] further observed significant increase of global circumferential and radial strain associated with aging, which was not observed for our cohort. Whether these discrepancies remain of technical or ethnical origin, remains undetermined. Moreover, controversy persists regarding age-related changes in circumferential, longitudinal, and radial strain rates, which however have been associated with overall low levels of reproducibility [10].

In summary, both recent research findings and the results of our present study align with the observation of an age-associated increase in global right ventricular strain. This underscores the importance of recognizing age-dependent variations in patient diagnostics, highlighting the need for tailored approaches in clinical practice.

The predictability of right ventricular global longitudinal strain by RV-GLSR diastole, age and sex, in respectively decreasing order of influence, was consolidated by the outcomes of the multiple linear regression analysis.

Consistent with findings of Li et al. [9] and Peng et al.[11], intra-class correlation was only moderate for interrater variability of the right ventricular longitudinal strain. These findings may result from the subjective input during contouring of the epicardium, particularly in the 4-chamber view. Moreover, the findings suggest a certain amount of rater-dependency. A consistent rater resulted in good to excellent levels of replicability consolidating CMR function for reliable assessment of all three right ventricular strain types as similarly observed recently [9].

Right ventricular strains can be diagnostically applied to various cardiopulmonary diseases [1–4, 22, 23]. Acknowledging age and sex specificity enhances a more patient-tailored diagnostic approach and offers the potential to identify more subtle, possibly subclinical alterations in cardiac wall motion. Utilizing CMR-feature tracking strain analysis, which accounts for spatial dimensions of contractile function, emerges as a dependable method for exploring physiological adaptations. This underscores the potential for widespread clinical utilization of right ventricular strains in diagnostic settings.

## Limitations

This study remains a retrospective single-center study unable to determine causal relationships. Furthermore, the study cohort is restricted to Caucasian study cohort, thus study data is not representative for or compared to other ethnicities. Furthermore, recruitment via public call may have been associated with selection bias, due to narrowed accessibility of the public call and the possibility of self-selection bias, attracting volunteers with similar characteristics and motivations. Although the physical activity of the participants could be a possible confounder for RV strain, these data were not statistically analyzed in this study (see Table 1). The primary deciding factor was the subjective perception and definition of a training session for physical activity by the participants. We therefore recommend using a much stricter definition of physical activity in further studies, for example, an app-based recording of 10.000 steps per day. In addition, other potential confounders such as lifestyle factors like diet, stress levels and/or socioeconomic status were not considered in this study. Lastly, the subgroup over 50 years of age contains fewer individuals mainly due to fewer subjects meeting the criteria for cardiac healthiness, limiting the generalizability of the study results.

## Conclusion

CMR-feature tracking provides fair reproducibility of age- and gender dependent normal right ventricular strain values. The present study results demonstrate that increased global longitudinal strains are associated with the female sex and advancing age within a Caucasian cohort of healthy subjects.

## Contributions

JE wrote the manuscript, analyzed the data, and does the interpretation of the data. HK designed the study, conducted the CMR imaging, analyzed the data, and revised the manuscript. OMW, WB, VP, OD and MP approved the content for publication and the final manuscript. All authors made substantial contributions to the article and approved the submitted version.

## Data Availability

The data that support the findings of this study are available on request from the corresponding author.

## Abbreviations

BMI: Body mass index
CMR: Cardiac magnetic resonance imaging
CoV: Coefficient of variation
FT: Feature tracking
GLS: Global longitudinal strain
ICC: Intra-class correlation coefficient
RV: Right ventricular
SD: Standard deviation
